# 3-[(*E*)-1-(Benzyl­oxyimino)­eth­yl]-7-(3-methyl­but-2-en­yloxy)-2*H*-chromen-2-one

**DOI:** 10.1107/S1600536811019015

**Published:** 2011-05-25

**Authors:** Hui Wang, Li-juan He, Wei-hua Zheng, Hua-can Song

**Affiliations:** a School of Chemistry and Environment, South China Normal University, Guangzhou 510006, People’s Republic of China; b School of Chemistry and Chemical Engineering, Sun Yat-sen University, Guangzhou 510275, People’s Republic of China

## Abstract

In the title compound, C_23_H_23_NO_4_, the dihedral angle beween the chromen-2-one ring system and the benzene ring is 69.73 (10)° and the mol­ecule adopts an *E* conformation with respect to the C=N double bond. In the crystal, inversion dimers linked by pairs of C—H⋯O hydrogen bonds occur, generating *R*_2_^2^(12) loops.

## Related literature

For background to the use of Schiff bases as chemosensors, see: Li *et al.* (2009 ▶).
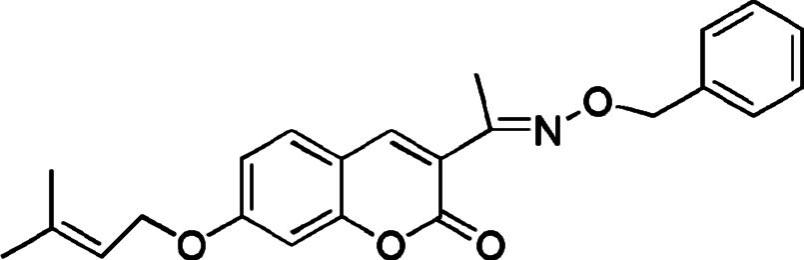


## Experimental

### 

#### Crystal data

C_23_H_23_NO_4_
*M* *_r_* = 377.42
Triclinic, 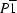

*a* = 7.3038 (19) Å
*b* = 11.467 (3) Å
*c* = 12.184 (3) Å
α = 92.368 (3)°
β = 92.067 (3)°
γ = 102.340 (3)°
*V* = 995.0 (5) Å^3^
*Z* = 2
Mo *K*α radiation
μ = 0.09 mm^−1^
*T* = 273 K
0.26 × 0.18 × 0.16 mm

#### Data collection

Bruker APEXII CCD diffractometer
Absorption correction: multi-scan (*SADABS*; Bruker, 2004) ▶ *T*_min_ = 0.978, *T*_max_ = 0.986
5604 measured reflections
3912 independent reflections
2341 reflections with *I* > 2σ(*I*)
*R*_int_ = 0.032

#### Refinement

*R*[*F* ^2^ > 2σ(*F* ^2^)] = 0.052
*wR*(*F* ^2^) = 0.155
*S* = 1.01
3912 reflections
257 parameters
H-atom parameters constrained
Δρ_max_ = 0.18 e Å^−3^
Δρ_min_ = −0.19 e Å^−3^

### 

Data collection: *APEX2* (Bruker, 2004) ▶; cell refinement: *SAINT* (Bruker, 2004) ▶; data reduction: *SAINT* ▶; program(s) used to solve structure: *SHELXS97* (Sheldrick, 2008 ▶); program(s) used to refine structure: *SHELXL97* (Sheldrick, 2008 ▶); molecular graphics: *SHELXTL* (Sheldrick, 2008 ▶); software used to prepare material for publication: *SHELXL97*.

## Supplementary Material

Crystal structure: contains datablocks global, I. DOI: 10.1107/S1600536811019015/hb5875sup1.cif

Structure factors: contains datablocks I. DOI: 10.1107/S1600536811019015/hb5875Isup2.hkl

Supplementary material file. DOI: 10.1107/S1600536811019015/hb5875Isup3.cml

Additional supplementary materials:  crystallographic information; 3D view; checkCIF report

## Figures and Tables

**Table 1:** Hydrogen-bond geometry (Å, °)

*D*—H⋯*A*	*D*—H	H⋯*A*	*D*⋯*A*	*D*—H⋯*A*
C8—H8⋯O2^i^	0.93	2.43	3.338 (3)	167

Symmetry code: (i) 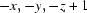.

**Fig. 1.**
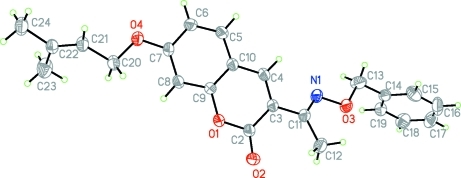
The molecular structure of the title compound showing the atom numbering scheme. Displacement ellipsoids are drawn at the 50% probability level.

**Fig. 2.**
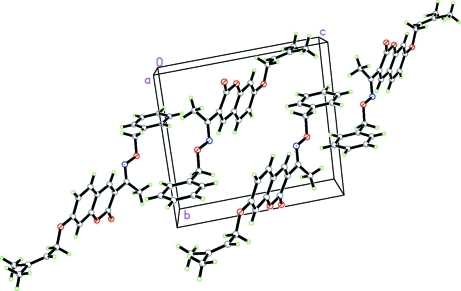
Crystal packing in the title compound.

**Fig. 3.**
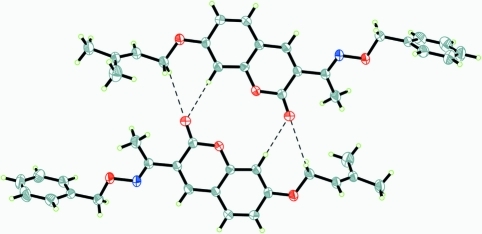
Part of the crystal structure of the title compound showing weak C—H···O hydrogen bonds as dashed lines.

**Fig. 4.**
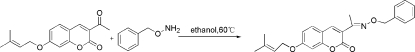
The formation of the title compound.

## supplementary crystallographic information

### Comment

Coumarin-derived Schiff bases have attracted attention as colorimetric chemosensors (Li *et al.*, 2009). Herein, we report the crystal structure of the title compound, (I), Fig. 1, obtained by the reaction of 3-acetyl-7-(3-methylbut-2-enyloxy)-2*H*- chromen-2-one with benzyloxy-amine.   Inversion dimers occur in the crystal, being linked by pairs of C—H···O hydrogen bonds (Table 1).

### Experimental

A mixture of 3-acetyl-7-(3-methylbut-2-enyloxy)-2*H*-chromen-2-one (1 mmol) and benzyloxy- amine hydrochloride (1.2 mmol) in ethanol (15 ml) was heated at 313 K for 0.5 h, the solution pH was then maintained at a value of 7 by the addition of sodium carbonate(0.5 mmol). The reaction mixture was refluxed for 10 h at 333 K (monitored by TLC). After completion of the reaction, the solvent was removed under a vacuum. The crude product was purified by chromatography (ethyl acetate: petroleum ether = 3:1). The eluate was evaporated to give the title compound as colourless blocks (279 mg, 74%; m. p. 372–374 K). ESI-MS (m / *z*): [(*M*+Na)+] 400, [(*M*+H)+] 378; IR (KBr, cm-1) 3065, 2971, 2877, 1722, 1603, 1491, 1458, 1357, 1259, 1216, 1129, 981, 923, 772; 1H NMR(400 MHz, CDCl3, TMS) delta 7.79 (s, 1H), 7.40 (d, 1H, J = 8.4 Hz), 7.31–7.36 (m, 5H), 6.83 (dd, 1H, J = 2.8, 8.4 Hz), 6.79(d 1H, J = 2.8 Hz), 5.45–5.50 (m, 1H), 5.23 (s, 2H), 4.56(d, 2H, J = 6.8 Hz), 2.27 (s, 3H), 1.80 (s, 3H), 1.76 (s, 3H); 13 C NMR (100 MHz, CDCl3) delta 162.45, 160.08, 155.90, 154.33, 141.40, 137.72, 129.49, 128.96, 128.41, 128.33, 127.97, 127.84,127.42, 121.30, 118.59, 113.57, 112.43, 101.08, 76.21, 65.46, 25.83,18.30,14.61.

### Crystal data

**Table:**

C_23_H_23_NO_4_	*Z* = 2
*M**_r_* = 377.42	*F*(000) = 430.0
Triclinic, *P*1	*D*_x_ = 1.260 Mg m^−^^3^
Hall symbol: -P 1	Mo *K*α radiation, λ = 0.71073 Å
*a* = 7.3038 (19) Å	Cell parameters from 1391 reflections
*b* = 11.467 (3) Å	θ = 2.4–24.9°
*c* = 12.184 (3) Å	µ = 0.09 mm^−^^1^
α = 92.368 (3)°	*T* = 273 K
β = 92.067 (3)°	Block, colorless
γ = 102.340 (3)°	0.26 × 0.18 × 0.16 mm
*V* = 995.0 (5) Å^3^	

### Data collection

**Table:**

Bruker APEXII CCD diffractometer	3912 independent reflections
Radiation source: fine-focus sealed tube	2341 reflections with *I* > 2σ(*I*)
graphite	*R*_int_ = 0.032
φ and ω scans	θ_max_ = 26.3°, θ_min_ = 1.8°
Absorption correction: multi-scan (*SADABS*; Bruker, 2004)	*h* = −9→8
*T*_min_ = 0.978, *T*_max_ = 0.986	*k* = −10→14
5604 measured reflections	*l* = −14→15

### Refinement

**Table:**

Refinement on *F*^2^	Secondary atom site location: difference Fourier map
Least-squares matrix: full	Hydrogen site location: inferred from neighbouring sites
*R*[*F*^2^ > 2σ(*F*^2^)] = 0.052	H-atom parameters constrained
*wR*(*F*^2^) = 0.155	*w* = 1/[σ^2^(*F*_o_^2^) + (0.0644*P*)^2^ + 0.1016*P*] where *P* = (*F*_o_^2^ + 2*F*_c_^2^)/3
*S* = 1.01	(Δ/σ)_max_ = 0.028
3912 reflections	Δρ_max_ = 0.18 e Å^−^^3^
257 parameters	Δρ_min_ = −0.19 e Å^−^^3^
0 restraints	Extinction correction: *SHELXL97* (Sheldrick, 2008), Fc^*^=kFc[1+0.001xFc^2^λ^3^/sin(2θ)]^-1/4^
Primary atom site location: structure-invariant direct methods	Extinction coefficient: 0.049 (5)

### Special details

**Table:**

Geometry. All e.s.d.'s (except the e.s.d. in the dihedral angle between two l.s. planes) are estimated using the full covariance matrix. The cell e.s.d.'s are taken into account individually in the estimation of e.s.d.'s in distances, angles and torsion angles; correlations between e.s.d.'s in cell parameters are only used when they are defined by crystal symmetry. An approximate (isotropic) treatment of cell e.s.d.'s is used for estimating e.s.d.'s involving l.s. planes.
Refinement. Refinement of *F*^2^ against ALL reflections. The weighted *R*-factor *wR* and goodness of fit *S* are based on *F*^2^, conventional *R*-factors *R* are based on *F*, with *F* set to zero for negative *F*^2^. The threshold expression of *F*^2^ > σ(*F*^2^) is used only for calculating *R*-factors(gt) *etc*. and is not relevant to the choice of reflections for refinement. *R*-factors based on *F*^2^ are statistically about twice as large as those based on *F*, and *R*- factors based on ALL data will be even larger.

### Fractional atomic coordinates and isotropic or equivalent isotropic displacement parameters (Å^2^)

**Table:**

	*x*	*y*	*z*	*U*_iso_*/*U*_eq_	
N1	0.4410 (2)	0.43803 (16)	0.26381 (14)	0.0548 (5)	
O3	0.59306 (19)	0.46642 (13)	0.19438 (12)	0.0611 (4)	
O2	0.3010 (2)	0.10766 (13)	0.38943 (12)	0.0622 (4)	
O1	0.04470 (18)	0.14660 (12)	0.45666 (10)	0.0492 (4)	
O4	−0.50894 (19)	0.21552 (13)	0.61925 (11)	0.0558 (4)	
C19	0.9868 (3)	0.6503 (2)	0.19649 (18)	0.0627 (6)	
H19	0.9821	0.6483	0.2726	0.075*	
C18	1.1555 (3)	0.6776 (2)	0.1505 (2)	0.0776 (8)	
H18	1.2648	0.6934	0.1952	0.093*	
C17	1.1667 (4)	0.6820 (3)	0.0413 (3)	0.0852 (9)	
H17	1.2835	0.7011	0.0107	0.102*	
C16	1.0072 (5)	0.6584 (3)	−0.0255 (2)	0.0898 (9)	
H16	1.0149	0.6616	−0.1014	0.108*	
C15	0.8327 (4)	0.6296 (2)	0.0215 (2)	0.0743 (7)	
H15	0.7235	0.6132	−0.0232	0.089*	
C14	0.8222 (3)	0.62545 (18)	0.13341 (18)	0.0539 (6)	
C13	0.6388 (4)	0.5931 (2)	0.1861 (3)	0.0872 (9)	
H13B	0.6471	0.6325	0.2587	0.105*	
H13A	0.5422	0.6184	0.1423	0.105*	
C11	0.3842 (3)	0.32488 (19)	0.26814 (16)	0.0485 (5)	
C12	0.4680 (4)	0.2348 (2)	0.2070 (2)	0.0742 (7)	
H12C	0.5063	0.2642	0.1369	0.111*	
H12A	0.3765	0.1611	0.1961	0.111*	
H12B	0.5750	0.2214	0.2486	0.111*	
C3	0.2208 (3)	0.28938 (17)	0.33767 (15)	0.0449 (5)	
C2	0.1984 (3)	0.17731 (18)	0.39329 (15)	0.0462 (5)	
C9	−0.0825 (3)	0.21796 (17)	0.47168 (14)	0.0418 (5)	
C4	0.0943 (3)	0.35857 (18)	0.35299 (15)	0.0481 (5)	
H4	0.1091	0.4300	0.3175	0.058*	
C10	−0.0610 (3)	0.32596 (17)	0.42160 (15)	0.0437 (5)	
C5	−0.1943 (3)	0.39482 (19)	0.44254 (17)	0.0530 (5)	
H5	−0.1839	0.4680	0.4104	0.064*	
C6	−0.3389 (3)	0.35666 (19)	0.50912 (17)	0.0529 (5)	
H6	−0.4246	0.4042	0.5231	0.064*	
C8	−0.2289 (3)	0.17562 (18)	0.53828 (15)	0.0460 (5)	
H8	−0.2400	0.1022	0.5699	0.055*	
C7	−0.3581 (3)	0.24560 (18)	0.55642 (15)	0.0458 (5)	
C20	−0.5503 (3)	0.0961 (2)	0.65844 (17)	0.0564 (6)	
H20B	−0.4576	0.0878	0.7150	0.068*	
H20A	−0.5481	0.0381	0.5985	0.068*	
C21	−0.7401 (3)	0.07576 (19)	0.70411 (16)	0.0536 (5)	
H21	−0.8390	0.0794	0.6550	0.064*	
C22	−0.7839 (3)	0.05328 (19)	0.80605 (16)	0.0516 (5)	
C24	−0.6452 (4)	0.0473 (3)	0.89720 (19)	0.0908 (9)	
H24C	−0.5213	0.0617	0.8696	0.136*	
H24A	−0.6738	−0.0304	0.9268	0.136*	
H24B	−0.6510	0.1069	0.9539	0.136*	
C23	−0.9843 (3)	0.0309 (2)	0.8392 (2)	0.0740 (7)	
H23B	−1.0643	0.0390	0.7770	0.111*	
H23C	−0.9965	0.0879	0.8969	0.111*	
H23A	−1.0200	−0.0484	0.8648	0.111*	

### Atomic displacement parameters (Å^2^)

**Table:**

	*U*^11^	*U*^22^	*U*^33^	*U*^12^	*U*^13^	*U*^23^
N1	0.0458 (10)	0.0545 (12)	0.0642 (11)	0.0066 (9)	0.0195 (8)	0.0119 (9)
O3	0.0547 (9)	0.0495 (10)	0.0799 (10)	0.0063 (7)	0.0285 (8)	0.0151 (7)
O2	0.0598 (9)	0.0552 (10)	0.0791 (10)	0.0235 (8)	0.0188 (8)	0.0198 (8)
O1	0.0493 (8)	0.0467 (9)	0.0545 (8)	0.0125 (7)	0.0135 (6)	0.0161 (6)
O4	0.0584 (9)	0.0500 (9)	0.0602 (9)	0.0102 (7)	0.0212 (7)	0.0094 (7)
C19	0.0675 (15)	0.0652 (16)	0.0569 (13)	0.0157 (13)	0.0105 (11)	0.0086 (11)
C18	0.0535 (15)	0.089 (2)	0.0876 (19)	0.0105 (14)	0.0042 (13)	0.0007 (15)
C17	0.0681 (18)	0.089 (2)	0.096 (2)	0.0059 (15)	0.0369 (16)	0.0044 (16)
C16	0.122 (3)	0.093 (2)	0.0524 (15)	0.0134 (19)	0.0287 (17)	0.0138 (14)
C15	0.0760 (18)	0.0710 (18)	0.0716 (16)	0.0083 (14)	−0.0149 (13)	0.0084 (13)
C14	0.0487 (13)	0.0434 (13)	0.0715 (14)	0.0097 (10)	0.0176 (11)	0.0137 (10)
C13	0.0692 (17)	0.0507 (16)	0.150 (3)	0.0176 (13)	0.0500 (17)	0.0331 (16)
C11	0.0480 (12)	0.0480 (13)	0.0491 (11)	0.0080 (10)	0.0081 (9)	0.0058 (9)
C12	0.0839 (18)	0.0575 (16)	0.0832 (17)	0.0131 (13)	0.0391 (14)	0.0051 (13)
C3	0.0458 (11)	0.0430 (12)	0.0453 (10)	0.0064 (9)	0.0075 (8)	0.0072 (9)
C2	0.0449 (11)	0.0469 (13)	0.0467 (11)	0.0082 (10)	0.0057 (9)	0.0072 (9)
C9	0.0435 (11)	0.0416 (12)	0.0412 (10)	0.0108 (9)	0.0031 (8)	0.0047 (8)
C4	0.0508 (12)	0.0435 (12)	0.0489 (11)	0.0060 (10)	0.0046 (9)	0.0111 (9)
C10	0.0431 (11)	0.0433 (12)	0.0436 (10)	0.0059 (9)	0.0032 (8)	0.0075 (9)
C5	0.0536 (13)	0.0454 (13)	0.0616 (13)	0.0110 (10)	0.0085 (10)	0.0146 (10)
C6	0.0519 (12)	0.0448 (13)	0.0649 (13)	0.0144 (10)	0.0097 (10)	0.0085 (10)
C8	0.0511 (12)	0.0427 (12)	0.0436 (10)	0.0069 (10)	0.0064 (9)	0.0084 (9)
C7	0.0465 (11)	0.0465 (12)	0.0425 (10)	0.0042 (9)	0.0101 (8)	0.0046 (9)
C20	0.0637 (14)	0.0540 (14)	0.0522 (12)	0.0108 (11)	0.0156 (10)	0.0106 (10)
C21	0.0486 (12)	0.0603 (14)	0.0501 (12)	0.0054 (10)	0.0072 (9)	0.0111 (10)
C22	0.0539 (13)	0.0507 (13)	0.0490 (11)	0.0066 (10)	0.0091 (9)	0.0062 (9)
C24	0.0810 (19)	0.136 (3)	0.0548 (14)	0.0192 (18)	0.0014 (13)	0.0171 (15)
C23	0.0656 (16)	0.0839 (19)	0.0709 (16)	0.0066 (14)	0.0209 (12)	0.0184 (13)

### Geometric parameters (Å, °)

**Table:**

N1—C11	1.279 (3)	C3—C4	1.354 (3)
N1—O3	1.4111 (19)	C3—C2	1.459 (3)
O3—C13	1.428 (3)	C9—C8	1.382 (2)
O2—C2	1.207 (2)	C9—C10	1.385 (3)
O1—C9	1.375 (2)	C4—C10	1.428 (3)
O1—C2	1.377 (2)	C4—H4	0.9300
O4—C7	1.356 (2)	C10—C5	1.402 (3)
O4—C20	1.443 (2)	C5—C6	1.363 (3)
C19—C18	1.353 (3)	C5—H5	0.9300
C19—C14	1.373 (3)	C6—C7	1.401 (3)
C19—H19	0.9300	C6—H6	0.9300
C18—C17	1.338 (3)	C8—C7	1.381 (3)
C18—H18	0.9300	C8—H8	0.9300
C17—C16	1.368 (4)	C20—C21	1.488 (3)
C17—H17	0.9300	C20—H20B	0.9700
C16—C15	1.396 (4)	C20—H20A	0.9700
C16—H16	0.9300	C21—C22	1.316 (3)
C15—C14	1.371 (3)	C21—H21	0.9300
C15—H15	0.9300	C22—C24	1.490 (3)
C14—C13	1.488 (3)	C22—C23	1.504 (3)
C13—H13B	0.9700	C24—H24C	0.9600
C13—H13A	0.9700	C24—H24A	0.9600
C11—C3	1.483 (3)	C24—H24B	0.9600
C11—C12	1.495 (3)	C23—H23B	0.9600
C12—H12C	0.9600	C23—H23C	0.9600
C12—H12A	0.9600	C23—H23A	0.9600
C12—H12B	0.9600		
			
C11—N1—O3	111.18 (16)	O1—C9—C10	120.44 (16)
N1—O3—C13	108.13 (15)	C8—C9—C10	123.15 (18)
C9—O1—C2	123.03 (15)	C3—C4—C10	122.46 (18)
C7—O4—C20	117.49 (15)	C3—C4—H4	118.8
C18—C19—C14	121.5 (2)	C10—C4—H4	118.8
C18—C19—H19	119.2	C9—C10—C5	117.12 (17)
C14—C19—H19	119.2	C9—C10—C4	117.73 (18)
C17—C18—C19	120.6 (3)	C5—C10—C4	125.15 (18)
C17—C18—H18	119.7	C6—C5—C10	121.29 (19)
C19—C18—H18	119.7	C6—C5—H5	119.4
C18—C17—C16	120.4 (2)	C10—C5—H5	119.4
C18—C17—H17	119.8	C5—C6—C7	119.89 (19)
C16—C17—H17	119.8	C5—C6—H6	120.1
C17—C16—C15	119.2 (2)	C7—C6—H6	120.1
C17—C16—H16	120.4	C7—C8—C9	118.06 (18)
C15—C16—H16	120.4	C7—C8—H8	121.0
C14—C15—C16	120.1 (2)	C9—C8—H8	121.0
C14—C15—H15	119.9	O4—C7—C8	124.63 (18)
C16—C15—H15	119.9	O4—C7—C6	114.90 (17)
C15—C14—C19	118.1 (2)	C8—C7—C6	120.47 (18)
C15—C14—C13	121.5 (2)	O4—C20—C21	107.58 (17)
C19—C14—C13	120.3 (2)	O4—C20—H20B	110.2
O3—C13—C14	108.12 (18)	C21—C20—H20B	110.2
O3—C13—H13B	110.1	O4—C20—H20A	110.2
C14—C13—H13B	110.1	C21—C20—H20A	110.2
O3—C13—H13A	110.1	H20B—C20—H20A	108.5
C14—C13—H13A	110.1	C22—C21—C20	127.4 (2)
H13B—C13—H13A	108.4	C22—C21—H21	116.3
N1—C11—C3	113.69 (18)	C20—C21—H21	116.3
N1—C11—C12	124.18 (19)	C21—C22—C24	124.4 (2)
C3—C11—C12	122.1 (2)	C21—C22—C23	121.23 (19)
C11—C12—H12C	109.5	C24—C22—C23	114.42 (18)
C11—C12—H12A	109.5	C22—C24—H24C	109.5
H12C—C12—H12A	109.5	C22—C24—H24A	109.5
C11—C12—H12B	109.5	H24C—C24—H24A	109.5
H12C—C12—H12B	109.5	C22—C24—H24B	109.5
H12A—C12—H12B	109.5	H24C—C24—H24B	109.5
C4—C3—C2	118.98 (17)	H24A—C24—H24B	109.5
C4—C3—C11	122.25 (18)	C22—C23—H23B	109.5
C2—C3—C11	118.75 (17)	C22—C23—H23C	109.5
O2—C2—O1	116.02 (17)	H23B—C23—H23C	109.5
O2—C2—C3	126.66 (18)	C22—C23—H23A	109.5
O1—C2—C3	117.31 (17)	H23B—C23—H23A	109.5
O1—C9—C8	116.41 (16)	H23C—C23—H23A	109.5

### Hydrogen-bond geometry (°)

**Table:**

*D*—H···*A*
—···

## References

[bb1] Bruker (2004). *APEX2*, *SAINT* and *SADABS* Bruker AXS Inc., Madison, Wisconsin, USA.

[bb2] Li, H. Y., Gao, S. & Xi, Z. (2009). *Inorg. Chem. Commun.* **12**, 300–303.

[bb3] Sheldrick, G. M. (2008). *Acta Cryst.* A**64**, 112–122. 10.1107/S0108767307043930 18156677

